# Predicting attitudes towards easing COVID-19 restrictions in the United States of America: The role of health concerns, demographic, political, and individual difference factors

**DOI:** 10.1371/journal.pone.0263128

**Published:** 2022-02-23

**Authors:** Adam Gerace, Gabrielle Rigney, Joel R. Anderson

**Affiliations:** 1 College of Psychology, School of Health, Medical and Applied Sciences, CQUniversity, Adelaide, South Australia, Australia; 2 School of Psychology, Australian Catholic University, Fitzroy, Victoria, Australia; 3 Australian Research Centre in Sex, Health and Society, La Trobe University, Bundoora, Victoria, Australia; Konkuk University, REPUBLIC OF KOREA

## Abstract

Despite rising cases of COVID-19 in the United States of America, several states are easing restrictions (e.g., relaxing physical distancing requirements, reopening businesses) that were imposed to limit community transmission of the virus. Individuals hold differing opinions regarding whether restrictions should continue to be imposed or lifted, evidenced, for example, by debate and protests regarding reopening of businesses and venues. Health and social psychological research suggest that perceptions of COVID-19related risk, experiences of the virus, and individual difference factors can help explain individuals’ attitudes towards health initiatives and their tendency to be persuaded towards a specific course of action. The purpose of this study was to investigate what factors influence support or opposition to easing COVID-19-related restrictions. A sample of 350 United States citizens, responding to an anonymous survey, were asked about the extent to which they support/oppose easing of COVID-19-related restrictions, both generally and in relation to specific restrictions. Respondents completed measures of their experiences of COVID-19, individual difference factors, and demographic variables, including political affiliation and degree of social and economic conservatism. In a series of regression analyses, significant demographic predictors of support or opposition for easing restrictions were gender, age, ethnicity, and education, with political affiliation and degree of social and economic conservatism also predicting attitudes. Experiences related to COVID-19 that predicted attitudes were concerns for self and family, perceptions of threat posed by the virus, perceived ability to adhere to restrictions, willingness to take government direction, and belief in COVID-19-related conspiracy theories. At an individual differences level, uncertainty avoidance, collectivism, long-term orientation, masculinity, empathic concern, personal distress, reactance, and general conspiracy theory beliefs all significantly precited attitudes to easing restrictions. Understanding the factors that help explain attitudes towards COVID-19 restrictions can inform how best to position health messaging and initiatives going forward, particularly as states or countries open borders.

## Introduction

In the United States of America, as of 30 November 2021, there had been 48,497,243 COVID-19 cases, with 780,131 deaths [1]. The United States has recorded both the highest number of COVID-19 cases worldwide and the highest number of deaths [2]. While confirmed cases were decreasing mid-2021 and amongst the lowest since the early months of the pandemic [3], and by the end of November approximately 58% of the population were fully vaccinated [4], new cases remain high with (as of 30 November) over 580,000 confirmed cases in the last seven days [5]. COVID-19 deaths in the United States have surpassed those of the 1918–1919 Spanish flu pandemic [6].

Despite the ongoing threat of the virus, there have been easing of restrictions in most states with a roadmap to reopening across the country [7]. The implementation of restrictions to reduce spread of the virus has been accompanied by considerable debate and public calls to ease restrictions and eliminate physical distancing recommendations, including protests both in the United States and worldwide [8]. There is ongoing research on the physical effects of the COVID-19 virus (e.g., [9]), psychological sequelae such as depression and anxiety (e.g., [10, 11]), and both the positive and negative effects of the pandemic on health behaviors, lifestyle, work, and relationships (e.g., [12–17]). However, research on what predicts people’s attitudes towards COVID-19-related restrictions and their motivation to adhere to such recommendations is in its infancy. While the COVID-19 pandemic is a relatively new health crisis, social and health psychology perspectives may be useful in understanding responses by American citizens to restrictions related to COVID-19. The purpose of this study, therefore, is to investigate what factors influence a person’s support or opposition to easing COVID-19-related restrictions.

### Background

COVID-19 has posed physical, economic, social, and psychological threats for individuals and communities, including threats to physical health and psychological security. Psychological perspectives can help inform understanding of responses to threat [18, 19], and consequently provide support for restrictions put in place to reduce infection and transmission. Common restrictions that have been implemented worldwide include physical or social distancing, mandated wearing of masks, work-from-home orders, and limits on travel. These restrictions have been implemented to bring about changes to human behavior. Transmission of the COVID-19 virus occurs through human behavior, and therefore having a greater understanding of key determinants of human behavior throughout a pandemic is critical.

Within health psychology, previous research established demographic and attitudinal determinants of protective behaviors during the SARS virus pandemic that occurred in the early 2000s. A systematic review by Bish and Michie [20] of 26 papers identified that perceptions of threat and the severity of the threat to oneself, and others, drive decision-making surrounding health-related activity. The review also found evidence that state anxiety and trust in authorities were two key factors associated with behavior during a pandemic. Based on the health psychology literature (e.g., [21–26]), *perceptions and experiences of COVID-19*, including perceptions of likelihood of contracting the virus (e.g., threat), personal exposure to the virus (e.g., knowing others who have contracted the virus), beliefs regarding outcomes of contracting the virus (e.g., severity), and emotions involved when considering the virus (e.g., worry) are factors that may help predict individuals’ support or opposition for easing COVID-19-related restrictions. Indeed, emerging worldwide research has supported the role of personal experiences of the COVID-19 virus in predicting perceptions of personal risk posed by the pandemic [27] and engagement in actual protective behaviors [28].

Health-decision making, such as engagement in protective behaviors, is based on exposure to information regarding illness, processing of that information, and belief in the appropriateness of responses to address the threat [29]. This may include judgments regarding the extent to which information sources can be trusted or believed [30]. In the case of COVID-19, actual knowledge of restrictions [31] and trust in information related to COVID-19 (e.g., from public health professionals; [31–33]) have been investigated for their relationship to adherence to recommendations and requirements.

Two types of trust relevant in this case are *trust in government* (e.g., [34, 35]) and *conspiracy theory beliefs* regarding powerful others (e.g., [36]). Not surprisingly, increased trust in government has been correlated with adoption of COVID-19 health-related behaviors, with governments that are more trusted being those where citizens perceive COVID-19 messaging is clear and that the government is organized in response to the pandemic [37]. Related to trust in government is actual *political affiliation*, where political affiliation (e.g., affiliation with the governing party or support of particular politicians) may influence perceptions of COVID-19 and restrictions. In four studies, Conway et al. [38] investigated mediators of the relationship between conservatism and lesser perceptions of threat posed by the virus. Factors including resistance (reactance) to and lesser support for government restrictions predicted lessened threat perceptions and mediated the conservatism-threat relationship, while factors including personal COVID-19 experience or consumption of partisan messaging (e.g., news) did not. The researchers interpreted these findings as reflecting motivations based on interests (e.g., to limit political interference) leading to downplaying of virus severity. Indeed, beyond political views, it is likely that those who have felt more socially or economically disadvantaged by COVID-19 may be more likely to want to ease restrictions.

In the case of conspiracy beliefs, greater belief in COVID-19 conspiracy theories is associated with lesser support of restrictions and lesser institutional trust of authorities (e.g., police, politicians, and health professionals; [39]). Indeed, Individuals in the United States who hold greater conspiracy beliefs related to the virus perceive lesser harm, engage in fewer protective behaviors, and have less intention to be vaccinated [40]. They also tend to rely more on conservative media and hold more conservative political ideologies [40], as well as have an increased distrust in experts [41].

At a wider level, individual difference factors, including attitudes regarding oneself and others, can usefully explain attitudes to COVID-19 restrictions. One theory that may be particularly relevant is Geert Hofstede’s [42] cultural dimensions theory. The theory, which has undergone revision over decades, suggests that individuals within a culture differ on six dimensions ([43]; for reviews see [44, 45]). These are (1) *uncertainty avoidance*, which is the degree of stress or threat felt when faced with unknowns of the future; (2) *power distance*, which involves support for maintaining hierarchies between citizens within society; (3) *masculinity*, which is a focus on competition and achievement; (4) *individualism versus collectivism*, which is the perceived degree of interdependence between self and others; (5) *long-term versus short-term orientation*, which involves attitudes towards tradition and social change as well as focus on longer-term versus shorter-term goals; and (6) *indulgence versus restraint*, which is degree of restraint in gratifying needs.

The cultural dimensions theory is based on data that uses an indirect values inference approach (i.e., secondary national-level data is used to ascribe characteristics based on cultural groupings, as opposed to collecting new data from cultural group members; [46]). While the theory itself is based on nation-level data, these dimensions may help explain people’s attitudes to easing or not easing restrictions in several ways. It can be argued that a response to COVID-19 depends on a need to focus on long-term rather than short-term goals (e.g., ‘flattening the curve’ vs. quickly ‘reopening’ society), restraint (e.g., adhering to social distancing despite negative effects), managing anxiety and uncertainty, lesser focus on competition, and a focus on others and the ‘greater good’ even if risk from the virus is lower for oneself. Moreover, the psychological processes or traits underlying many of these dimensions are reflective in psychological concepts investigated at the individual level, including uncertainty avoidance [47], social dominance orientation [48], cultural orientation [49], desirability for control [50], sex roles [51, 52], and long-term orientation [53].

Several studies have begun to investigate the role of some of these factors in understanding individuals’ COVID-19 responses. At a national level, Dheer et al. [54] found that nations that were more collectivist than individualistic, were higher rather than lower in power distance orientation, and were lower rather than higher in uncertainty avoidance, had lesser COVID-19 case growth. For the other factors, findings regarding masculinity versus femininity were more mixed and there were few significant relationships between case growth and long-term versus short-term orientation (indulgence/restraint was not measured). At the individual level, the concept of individualism versus collectivism has been particularly investigated for its utility in predicting psychological adjustment [55], perceptions of risk [27, 28, 55], and engaging in recommended prevention measures [56]. In these studies, more individualistic worldviews are associated with lesser perceptions of risk and use of prevention measures. However, the impact on attitudes towards lockdowns was not focused upon in these studies.

Another factor useful for understanding responses to restrictions is *empathy*. Empathy involves understanding the perspectives of others, particularly their negative experiences or plight, and feeling concern and care towards them [57]. Importantly, developing empathic concern for a needy other person, through the effortful process of apprehending their perspective, results in a motivation to help them [58]. Therefore, it is likely that those who are more predisposed to consider others’ situations, even if different to their own, and to experience empathic concern, would be more likely to want to help vulnerable others by maintaining restrictions. Indeed, in a study investigating empathic emotional reactions to others affected by COVID-19, participants with higher empathic concern for others, or those who were exposed to an elderly person affected by COVID-19, reported increased intended and actual behaviors related to physical distancing and mask wearing [59]. In another study, measures of perspective taking and empathic concern were found to significantly predict adherence to guidelines ([56]; for similar findings regarding perspective taking, see [60]).

Like empathy, the individual difference characteristic *need for cognition* involves effortful examination of information. Need for cognition is an individual’s tendency to engage in and enjoy such effortful information processing [61–63]. Such an approach to thinking is related to lesser dogmatism, decreased likelihood of ignoring or distorting information, and lesser need for predictability, certainty, and structure [63]. It may be argued that individuals higher in need for cognition would more closely examine COVID-19-related information, respond less to fear-related information, perceive the threat posed by large case numbers in the United States, and be more likely to approve of restrictions. Indeed, studies have found that need for cognition predicts increased perceptions of COVID-19 risk, but not other factors such as seeking specific news sources (e.g., television vs. social media; [64]) or panic buying [65].

By contrast, *reactance*, first conceptualized by Brehm [66], is the arousal of a motivational state to restore threatened or eliminated behavioral freedoms [67]. Individuals differ in the extent to which they are averse to giving up behavioral freedoms, and in their tendency to experience reactance in response to perceived threats [68]. Recent conceptions suggest that the concept involves anger and negative cognitions [69], and that reactance is higher when fear is aroused but cannot be reduced [70]. Reactance has been investigated for its potential to aid understanding of responses to health communication, with factors such as framing of the message (e.g., gain vs. loss), focus on self or others, and induced empathy important to understanding reactance responses [71]. Specific to COVID-19, lesser reactance has been found to predict future intentions to comply with COVID-19 safety recommendations (e.g., [60, 72]), and intentions to be vaccinated against the virus [73].

### Overview and aims

Based on the above-reviewed literature, the purpose of this study was to investigate these constructs as predictors of support (or opposition) for easing restrictions, such as ending enforced physical/social distancing and re-opening public spaces. The empirical contributions of this paper are two-fold. First, there are a range of constructs that are central to this paper for which there are no existing papers available. Thus, the first major aim is to develop a range of measures that can be used to tap the following: (a) COVID-19 Conspiracy Theories; (b) Indulgence; (c) General Attitudes to Easing COVID-19 Restrictions; and (d) Attitudes to Easing Specific COVID-19 Restrictions. Second, we used these new measures as a part of an online survey exploring ‘Perceptions of COVID-19 in the United States of America’. While the study is largely exploratory, based on previous health and social psychological research, as well as the emerging literature related to COVID-19, it was hypothesized that support for easing restrictions will be predicted by:

1. *Personal experiences of COVID-19*: specifically, lesser perceptions of risk and severity for self and family/friends of contracting COVID-19; greater perceptions of impact of COVID-19 on social and economic wellbeing; lesser perceptions of being able or willing to comply with restrictions; and greater beliefs in COVID-19 conspiracy theories.
2. *Political beliefs*: specifically, greater social and economic conservatism.
3. *Cultural values*: specifically, greater masculinity; lesser collectivism, power distance, uncertainty avoidance, long-term orientation, and self-restraint.
4. *Individual difference factors*: specifically, lesser empathy and need for cognition; lesser trust in government; and greater reactance and belief in general conspiracy theories.
5. *Demographic factors*: While not a primary focus of the research, demographic characteristics were also investigated. Based on emerging research on COVID-19 responses [e.g., 74–76], it was predicted greater support for easing restrictions would be associated with younger age, being male, lower educational attainment, and lower income.

## Method

### Participants

Based on a calculation conducted in G*Power, to detect a small effect (*f* = .20), with statistical power of 0.8 in a linear multiple regression, a sample size of at least 146 participants was required (α = .05). The calculation was based on a conservative estimate of effect given that, at the time of data collection, investigations in the area were only emerging making comparison with other studies difficult. A larger sample of 350 participants was sought as it was considered likely that some participant data would be excluded when screened during analysis for outliers or if participants did not complete a significant proportion of the survey, and because it was unknown how many factors might emerge during factor analysis on newly-devised measures. Participants residing in the United States were recruited via Prolific (an online participant recruitment platform). Individuals registered with Prolific provide demographic information that is used as part of the recruitment process. To obtain a representative sample, the option was selected to cross-stratify respondents by sex, age, and ethnicity, meaning that once a certain quota of respondents was reached, no more respondents meeting a particular criteria (e.g., age group) could complete the survey.

After data cleaning (described later in the paper), a final sample of 332 respondents were included in analyses. Respondents were asked a series of demographic questions regarding gender, age, ethnicity, highest level of education completed, household income in past 12 months, and residing state. Respondent demographics (frequencies and percentages) are presented in Table 1. Mean (*M*) age of the respondents was 45.42 years (*Standard Deviation (SD)* = 16.01; *Range* = 18–78 years). Forty-two of 50 states were represented, with states with at least 10 respondents reported in the table. There were roughly even proportions of male and female respondents, with the sample well-educated. Self-reported ethnicity was similar to proportions observed in the broader population of the United States.

**Table 1:** Respondent demographics.

	Frequency	%
*Gender*		
Female	168	50.60
Male	161	48.49
Non-binary or gender diverse	2	0.60
Prefer not to say	1	0.30
*State*		
New York	52	15.66
California	48	14.46
Florida	27	8.13
Texas	20	6.02
Georgia	11	3.31
Illinois	11	3.31
Massachusetts	10	3.01
Michigan	10	3.01
Washington	10	3.01
Other states	132	39.76
Missing	1	0.30
*Education*		
High school or less	34	10.24
Some college	77	23.19
Undergraduate degree	139	41.87
Postgraduate degree	82	24.70
*Household income*		
Less than $59,000	180	54.22
More than $59,000	152	45.78
*Ethnicity*		
White	225	67.77
Black	49	14.76
Asian	28	8.43
Hispanic	12	3.61
Other	18	5.42

### Materials

#### Attitudes to easing restrictions

The *General Attitudes to Easing COVID-19 Restrictions* Measure consisted of 10 items measuring respondents’ general attitudes towards easing COVID-19 restrictions. Items are included in Table 2. Items were completed on a Likert-type response scale from 1 (*strongly disagree*) to 5 (*strongly agree*).

**Table 2:** Item loadings for a forced single-factor model for general attitudes to easing COVID-19 restrictions items using Principal Axis Factoring.

Item	Factor
It is time to ‘get back to business’	.88
We should wait until COVID-19 cases have decreased more before easing restrictions (reversed)	.84
It is more important to reopen society than it is to completely eradicate COVID-19	.82
We should wait longer before easing restrictions (reversed)	.81
The dangers of reopening during COVID-19 have been overplayed by the media	.80
We are moving too fast in easing restrictions (reversed)	.80
It is important to ease restrictions so that people are not isolated from their families and friends	.79
It is important to ease restrictions so that people can go back to work/earn a living	.78
Maintaining restrictions (e.g., social distancing, keeping public places closes) is our best hope for limiting the number of people who catch COVID-19 (reversed)	.71
If people do the right thing (e.g., wash hands, sanitize), there is no problem with easing restrictions	.67

The *Attitudes to Easing Specific COVID-19 Restrictions* Measure consisted of 16 items measuring respondents’ opposition or support for easing of specific restrictions in their state. Respondents were instructed to respond to the items in the following way:

“*State governments in the United States have implemented several measures to try to reduce the spread of COVID-19*, *such as enforcing social distancing and closing public spaces and restaurants*. *Some states are easing restrictions that have been put in place (e*.*g*., *allowing people to meet in public) or ‘reopening’ venues and services*.

*Please indicate to what extent you support or oppose the government of your state easing restrictions*. *Some of these things may have already happened–for example*, *education institutions may have already opened in your state*. *Others may not have happened yet*. *Consider how much you support restrictions that have or will be lifted in your state*.”

Items are included in Table 3. Items were completed on a Likert-type response scale from 1 (*strongly oppose*) to 5 (*strongly favor*). Factor structures of both the general and specific measures were assessed prior to use.

**Table 3:** Item loadings for a forced single-factor model for specific attitudes to easing COVID-19 restrictions items using Principal Axis Factoring.

Item	Factor
Reopening entertainment venues (e.g., libraries theaters; bowling alleys; museums; casinos)	.86
Reopening gyms/fitness centers	.84
Reopening churches/places of worship	.83
Reopening personal care (e.g., hairdressers; nail salons; beauty parlors) salons	.81
Reopening bars and restaurants/cafes	.79
Reopening educational institutions (e.g., schools, universities)	.78
Allowing people to return to work in their offices	.78
Reopening retail stores	.76
Ending stay at home orders	.73
Easting requirements regarding domestic (within United States) travel	.70
Reopening outdoor recreation centers (e.g., pools, spas, beaches)	.69
Easing social distancing (e.g., 6 feet distance) requirements	.65
Allowing public gatherings of any size	.64
Easing requirements that people must wear masks while in public places	.63
Easing restrictions regarding international travel (traveling to/from United States to other countries)	.58
Allowing protests in public settings, such as the recent protests following the death of George Floyd*	.07

*Note*:

* Not included in final scale due to low factor loading.

#### Political views

Respondents reported their political views on social (“How would you characterize your political views about social issues?”) and economic (“How would you characterize your political views about economic issues?”) issues on two Likert-type response scales ranging from 1 (*Left-wing* [*progressive*]) to 7 (*Right-wing* [*conservative*]). They were also asked to indicate their political party identification (“In politics, as of today, do you consider yourself a Republican, a Democrat, or an independent?”).

Trust in government was measured using the *Trust in Government Scale* [35], 14-item scale measuring respondents’ perceptions of governments and politicians in relation to elements such as honesty, transparency, and understanding of constituents’ concerns. Example items include “Governments treat each group within society equally” and “Politicians tend to look after their own interests rather than trying to help others” (reversed). Respondents utilised a Likert-type response scale from 1 (*strongly disagree*) to 5 (*strongly agree*). Scores can range from 14 to 70, with higher scores indicating greater trust in government.

#### COVID-19 experiences and beliefs

First, respondents were asked whether they had been diagnosed with COVID-19, as well as if they had a family member/friend/loved one or an acquaintance who had been diagnosed with COVID-19 and, if so, how serious (1 = *Not at all serious/no symptoms*; 5 = *Extremely serious*) were symptoms for the particular person. If respondents knew multiple people who had been diagnosed with COVID-19, they were asked to report symptom severity for the person with the most serious symptoms.

Second, respondents completed a series of statements regarding their perceptions of their own risk of contracting COVID-19, severity of symptoms expected, and severity of symptoms expected should a love one contract the virus. Factor structure of the measure was assessed prior to use.

Next, respondents were asked about behavior during COVID-19, including adhering to restrictions (e.g., “I adhere to the COVID-19 restrictions recommended in my state” from 1 [*strongly disagree*] to 5 [*strongly agree*]), media engagement (e.g., “How often do you engage with media [e.g., TV, newspapers, internet sites] related to COVID-19?” from 1 [*never*] to 5 [*a great deal*]), wearing a mask in public (e.g., “How often do you wear a mask out in public?” from 1 [*never*] to 5 [*always*]), and perceptions of (then) President Trump’s handling of the COVID-19 situation (“How satisfied are you with President Trump’s handling of the COVID-19 situation in the United States?” from 1 [*very dissatisfied*] to 5 [*very satisfied*]).

Concerns about COVID-19-related outcomes were measured using the *COVID-19 Multifaceted Threat Scale* [77], a measure of respondents’ perceived threats about COVID-19 in three areas, each measured with a subscale consisting of 10 items: wellbeing threat–health and existential concerns (e.g., “I am worried because my mental health is being impacted”), social threat–relational and lifestyle concerns (e.g., “I am worried because I feel increasingly distant from my loved ones”), and material threat–supply and financial concerns (e.g., “I am worried because my financial situation is less stable”). Respondents indicated their threat concerns on a Likert-type response scale from 1 (*not at all worried*) to 7 (*extremely worried*). Scores on each subscale can range between 10 and 70, with higher scores reflecting greater perceived threat.

To measure *COVID-19 specific conspiracy beliefs*, six separate items were written specifically for this study. The items were generated based on beliefs that were being discussed on social media at the time. Specifically, we searched for beliefs that were specific to COVID-19 that met the definition of conspiracy beliefs (based on [36]) and synthesized them so that the six most-commonly identified arguments were turned into sentences that respondents could complete on a Likert-type response scale from 1 (*definitely not true*) to 5 (*definitely true*).

#### Cultural values

Hofstede’s dimensions of national culture were measured using the *Individual Cultural Values Scale* [78], an individual-difference level measure of five of the cultural dimensions using five subscales: *Power Distance* (5 items; e.g., “People in higher positions should not ask the opinions of people in lower positions too frequently”; score range 5–25); *Uncertainty Avoidance* (5 items; e.g., “Rules and regulations are important because they inform me of what is expected of me”; score range 5–25); *Collectivism* (6 items; e.g., “Individuals should only pursue their goals after considering the welfare of the group”; score range 6–30); *Long-Term Orientation* (6 items; e.g., “Going on resolutely in spite of opposition (Persistence)”; score range 6–30); and *Masculinity* (4 items, e.g., “It is more important for men to have a professional career than it is for women”; score range 4–20). Indulgence versus Restraint, which was not included in the measure (this is a more-recently added dimension), was measured in the present study utilising 4 items based on those used in Hofstede’s [79] Values Survey Module for national samples (e.g., “Keeping time for fun”). Respondents utilise a Likert-type response scale from 1 (*strongly disagree*) to 5 (*strongly agree*) for all but Long-Term Orientation and Indulgence/Restraint, which involved a 1 (*very unimportant*) to 5 (*very important*) response scale. Higher scores indicate greater uncertainty avoidance, collectivism, long-term orientation, masculinity, and indulgence, respectively.

#### Empathy

Empathy was measured using three seven-item subscales of the *Interpersonal Reactivity Index* (IRI; [80]): *Perspective-Taking* measures an individual’s propensity to take the perspectives of others (e.g., “I sometimes try to understand my friends better by imagining how things look from their perspective”); *Empathic Concern* measures tendency to experience feelings of concern, care and sympathy for others (e.g., “I often have tender, concerned feelings for people less fortunate than me”); and *Personal Distress* measures tendency towards distress-type reactions in emotional interpersonal or emergency situations (e.g., “Being in a tense emotional situation scares me”). Respondents utilise a Likert-type response scale from 0 (*does not describe me well*) to 4 (*describes me very well*). Scores on each subscale can range from 0 to 28, with higher scores indicating a greater disposition to experience the component of empathy.

#### Need for cognition

Need for cognition was measured using the *Need for Cognition Scale-6* [22], a six-item version of the Need for Cognition Scale [61] measuring disposition to prefer, engage in, and enjoy effortful thinking. An example item is “I like to have the responsibility of handling a situation that requires a lot of thinking”. Respondents utilise a Likert-type response scale from 1 (*extremely uncharacteristic of me*) to 5 (*extremely characteristic of me*). Scores can range from 5 to 30, with higher scores indicating a greater disposition to prefer effortful thinking.

#### Reactance

Reactance was measured using the *Hong Psychological Reactance Scale* [68], an 11-item version of the original scale [81] measuring motivation to restore threatened or lost freedoms [66, 82]. Example item are “Regulations trigger a sense of resistance in me” and “I become frustrated when I am unable to make free and independent decisions”. Respondents utilise a Likert-type response scale from 1 (*disagree completely*) to 5 (*agree completely*). Scores can range from 11 to 55, with higher scores indicating a greater disposition to experience reactance.

#### Conspiracy beliefs

Beliefs in conspiracy theories were measured using the *Generic Conspiracist Beliefs* [83] scale, a 15-item scale measuring individuals’ tendency towards explanations for events that stress conspiracy theories. Example items include, “Certain significant events have been the result of the activity of a small group who secretly manipulate world events” and “The spread of certain viruses and/or diseases is the result of the deliberate, concealed efforts of some organization”. Respondents utilise a Likert-type response scale from 1 (*definitely not true*) to 5 (*definitely true*). Scores can range from 15 to 75, with higher scores indicating a greater disposition to conspiracist ideation.

#### Socially desirable responding

Socially desirable responding was measured using the *Marlowe–Crowne Social Desirability Scale*, Form C [84] consisting of 13 items, including “No matter who I’m talking to, I’m always a good listener”. Respondents utilized a Likert-type response scale from 1 (*strongly disagree*) to 5 (*strongly agree*). Scores can range from 13 to 65, with higher scores indicating a greater disposition to socially desirable responding.

### Procedure

Study details were advertised on Prolific. Those interested in participating clicked on a URL in the study description to access the full study details via the Study Information Sheet. Respondents were provided with a project overview and description of what they would be required to do. They were also advised of the potential benefits and risks of participation, that participation was voluntary, that they could withdraw from the study prior to completion by closing their browser window, and that all collected data would be anonymous. They were also provided with the contact details of the first author and the University’s Ethics Committee if they had concerns and were also provided with a URL for a list of free telephone counseling national and state hotlines in the unlikely event of any distress. Potential respondents were advised that “Your consent to participate in this project will be obtained through your agreement to the Electronic Consent below”, which stated “Clicking on the ‘next’ (forward arrow) button below indicates that: (1) You have read the above information; (2) You voluntarily agree to participate; and You give your consent for the data you provide in the following survey to be used for the assessment and research purpose described above”. Such an approach to informed consent was based on ethical guidelines and principles in both the United States and in the researchers’ country [85, 86]. The study was approved by CQUniversity Human Research Ethics Committee (Application ID 0000022499). Since data collected was entirely anonymous and non-identifiable and respondents were not recruited from particular organisations requiring separate approvals, additional ethical approvals outside of the host University’s Ethics Committee were not required.

On clicking next, respondents were taken to the survey, which was hosted on the Qualtrics platform. Respondents completed demographic measures, followed by COVID-19 experience items/measures, including attitudes to easing restrictions measures, followed by the trait and individual difference measures. On completion of the survey, respondents were paid approximately USD$4.20.

Data was collected over a three-day period (31 July-2 August) in 2020.

## Results

### Data screening

Nine univariate outliers (-3.29 < *z*s > 3.29) were identified, with scores on these specific scales removed, but the rest of the respondents’ data retained. Data from 18 respondents were entirely deleted: six due to multivariate outliers (via Mahalanobis distance; *p* < .001), six due to respondents spending less than 10 minutes to complete the survey, and six due to respondents indicating that they had contracted COVID-19, which owing to the small number of respondents, could not be controlled for in analyses. The final sample for subsequent analyses consisted of 332 respondents.

### COVID-19 factors

#### Psychometric properties of new measures

Prior to main hypothesis testing, new measures were submitted to exploratory factor analysis. In all analyses reported below, Kaiser-Meyer-Olkin measures of sampling adequacy and Bartlett’s test of sphericity indicated the suitability of the data for factor analysis. Anti-image correlation matrices revealed that all measures of sampling adequacy for included items were above .60 [87]. For all analyses, parallel analysis, using a Monte Carlo analysis with 1,000 replications, was used, followed by Principal Axis Factoring (Direct Oblimin rotation). For individual items to be retained, they had to load at least .30 on a factor.

For both the *General Attitudes to Easing COVID-19 Restrictions* Measure and *Attitudes to Easing Specific COVID-19 Restrictions* Measure, parallel analysis suggested the extraction of one factor each (see Tables 2 and 3). Factor analyses for each of the new measures are presented in Tables 2–7.

**Table 4:** Item loadings for a forced single-factor model for COVID-19 conspiracy theories using Principal Axis Factoring.

Item	Factor
The government of the United States is being deliberately held back by the World Health Organisation	.83
The World Health Organisation have been giving preferential treatment to China over the United States	.82
The Chinese government destroyed medical records of early COVID-19 so that other countries would be unprepared	.77
COVID-19 disease originated in a laboratory	.77
Governments are using COVID-19 to pass laws aimed at controlling its people	.56
The Chinese government are withholding information from the United States government	.47

**Table 5:** Item loadings for a forced single-factor model for indulgence using Principal Axis Factoring.

Item	Factor
Being a happy person	.64
Not letting other people or circumstances stop you from doing what you want to do	.57
Keeping time free for fun	.47
Having few desires (moderation) [reversed]*	-.16

*Note*:

* Not included in final scale due to low factor loading.

**Table 6:** Item loadings for a forced three-factor model for perceptions of risk using Principal Axis Factoring.

Item	Factor 1 Own Severity	Factor 2 Other Risk	Factor 3 Own Risk
If I contracted COVID-19, I would be at higher risk for more severe symptoms (e.g., due to my age, pre-existing conditions)	.77		
I expect that I will be OK even if I do contract COVID-19 (reversed)	.72		
If I contracted COVID-19, it would be a serious problem for me	.67		
I am concerned that, if I contracted COVID-19, I might pass it on to a family member, friend, or loved one		-.81	
I am concerned about what would happen to a family member/friend/loved one if they contracted COVID-19		-.78	
If I contracted COVID-19, it would be a serious problem for someone I know (e.g., family member, friend)		-.73	
I have family members/friends/loved ones who would be at higher risk for more severe symptoms if they caught COVID-19 (e.g., due to age, pre-existing conditions)		-.64	
I often think about what would happen to me if I contracted COVID-19			.72
I am fearful of contracting COVID-19			.55
It is likely that I will contract COVID-19			.50
I am concerned about what will happen to me if I contracted COVID-19			.44
There is not a lot I can do to avoid catching COVID-19			.34

**Table 7:** Item loadings for a forced two-factor model for adherence to COVID-19 restrictions using Principal Axis Factoring.

Item	Factor 1 Adherence	Factor 2 Adherence Difficulty
I am confident in my ability to follow COVID-19 restrictions imposed by my state	.74	
I adhere to the COVID-19 restrictions recommended in my state	.72	
I am motivated to adhere to the COVID-19 restrictions imposed in my state	.69	
Guidelines in my state regarding restrictions are confusing to follow (reversed)*		.81
Recommendations made by health experts regarding COVID-19 are difficult to understand (reversed)*		.76
It is difficult to adhere to the COVID-19 restrictions imposed by my state (e.g., social distancing) (reversed)*		.53

*Note*:

* Items were reversed for factor analysis. For ease of interpretation, the original items (i.e., not reversed items) were totaled to create the subscale score.

### Descriptive and correlational data

#### Study scales

Table 8 contains the means (*M*), standard deviations (*SD*), and Cronbach’s alphas (*α*) of study scales. Mean scores on both easing restriction measures (general attitudes and attitudes to easing specific restrictions in one’s state) indicated that respondents were more opposed than supportive of easing restrictions. Respondents considered themselves to be of moderate risk for contracting COVID-19, and that there would be significant consequences for themselves and their loved ones should they contract the virus. They also indicated that they had significant concerns regarding COVID-19, although perceptions of threat (health and wellbeing, social, and material) were rated more moderately.

**Table 8:** Descriptive data for measures.

Measure	*M* (SD)	Cronbach’s α
Attitudes to Easing COVID-19 Restrictions	24.03 (9.88)	.94
Attitudes to Easing Specific COVID-19 Restrictions	36.91 (14.93)	.95
Trust in Government	30.01 (8.58)	.90
COVID-19 Concerns–Own Risk	15.43 (4.16)	.77
COVID-19 Concerns–Family Risk	16.09 (3.65)	.83
COVID-19 Concerns–Own Severity	10.53 (2.76)	.77
Adherence	13.51 (1.59)	.75
Adherence Difficulty	6.00 (2.50)	.76
COVID-19 Conspiracy Theories	17.42 (5.60)	.85
Threats–Wellbeing	35.42 (12.81)	.87
Threats–Social	35.36 (14.86)	.91
Threats–Material	36.91 (15.75)	.92
Power Distance	8.97 (3.68)	.81
Uncertainty Avoidance	20.54 (2.66)	.72
Collectivism	20.55 (4.80)	.87
Long-Term Orientation	25.22 (3.31)	.77
Masculinity	9.37 (4.05)	.83
Indulgence	12.31 (2.04)	.57
Perspective-Taking	19.73 (4.55)	.78
Empathic Concern	21.19 (5.02)	.83
Personal Distress	11.43 (5.44)	.79
Need for Cognition	21.63 (5.35)	.86
Reactance	28.67 (8.26)	.86
Conspiracy Theory Beliefs	39.56 (14.05)	.94
Social Desirability	41.39 (8.25)	.81

For individual difference variables, respondents were high on empathic responsiveness (perspective taking and empathic concern) and need for cognition, lower on personal distress, and moderate on reactance and social desirability concerns. For the cultural values dimensions, respondents were quite high on all measures except power distance and masculinity beliefs. Respondents indicated lesser trust in government, and they were lower on their espousing of both general and COVID-19-specific conspiracy beliefs.

Pearson’s correlation coefficients (*r*) indicate that general and specific attitudes to restrictions were highly correlated (*r* = .86, *p* < .001; see Table 9). Bivariate correlations with other variables were (not surprisingly) similar for these two measures, with the largest significant correlations involving an association between greater beliefs in COVID-19 conspiracy theories and increased support for easing restrictions on both measures, and associations between greater perceptions of severity of COVID-19 for self and others and lesser support for easing restrictions.

**Table 9:** Pearson’s correlation coefficients between variables.

	1	2	3	4	5	6	7	8	9	10	11	12	13	14	15	16	17	18	19	20	21	22	23	24
1. General Attitudes																								
2. Specific Attitudes	.86***																							
3. Trust Gov	.06	.04																						
4. Own Risk	-.27***	-.28***	-.05																					
5. Family	-.38***	-.35***	-.15**	.45***																				
6. Severity	-.38***	-.36***	-.10	.58***	.64***																			
7. Adhere	-.47***	-.45***	.07*	.19**	.25***	.26***																		
8. Diff adh	.34***	.33***	.03	.06	-.17**	-.06	-.40***																	
9. CVD Cons	.49***	.46***	-.17**	.07	-.10	-.07	-.39***	.35***																
10. Well	-.23***	-.26***	-.02	.70***	.39***	.49***	.08	.18**	.06															
11 Social	.09	.06	.08	.41***	.22***	.24***	-.08	.24***	.22***	.65***														
12. Mat	-.05	-.09	-.04	.54***	.30***	.34***	-.01	.27***	.20***	.67***	.55***													
13. Power	.25***	.21***	.22***	.11*	-.21***	-.01	-.15**	.30***	.24***	.12*	.18**	.16**												
14. UA	-.10	-.11*	.01	.18**	.18**	.14*	.13*	-.14*	.09	.18**	.23***	.20***	.09											
15. Collect	-.15**	-.13*	.15**	.12*	.20***	.17**	.17**	-.07	-.09	.19***	.26***	.15**	.23***	.46***										
16. LTO	.13*	.09	.07	.06	.08	.04	.07	-.13*	.13*	.05	.18**	.20***	-.03	.35***	.21***									
17. Mas	.41***	.36***	.12*	.09	-.17**	-.09	-.26***	.26***	.45***	.09	.29***	.23***	.56***	.21***	.20***	.20***								
18. Indulge	.10	.13*	-.05	-.02	-.02	-.08	.06	-.07	.09	.01	.17**	.04	-.003	.22***	.08	.37***	.19**							
19. PT	-.09	-.10	.04	-.04	.17**	.02	.16**	-.15**	-.06	-.04	.05	.06	-.23***	.20***	.21***	.27***	-.09	.26***						
20. EC	-.14*	-.16**	-.01	.02	.16**	.10	.25***	-.19**	-.16**	.02	.01	.07	-.30***	.19**	.12*	.24***	.23***	.28***	.55***					
21. PD	-.12*	-.13*	.05	.34***	.17**	.23***	.02	.18**	.04	.43***	.34***	.32***	.14*	.21***	.13*	-.11	.06	-.07	-.22***	-.06				
22. NFC	-.05	-.08	.03	-.06	.07	.02	.13*	-.09	-.11*	.02	.11*	-.02	-.11	-.05	.13*	.17**	-.09	.01	.25***	.14*	-.31***			
23. React	.09	.15**	-.10	.22***	.07	.14*	-.20***	.25***	.25***	.25***	.22***	.23***	.21***	-.10	-.14*	-.07	.21***	.01	-.22***	-.24***	-16**	.02		
24. Conspir	.30***	.30***	-.20***	.10	-.07	-.03	-.35***	.25***	.69***	.18**	.28***	.32***	.27***	.13*	.04	.11	.47***	.18**	-.01	-.12*	.09	-.16**	.29***	
25. SD	.11*	.09	.11	-.34***	-.28***	-.28***	.05	-.14**	.002	-.36***	-.24***	-.23***	-.10	.03	.004	.19**	.04	.12*	.29***	.31***	-.35***	.15**	-.45***	-.07

*Note*:

*** *p* < .001,

** *p* < .01,

* *p* < .05.

Trust Gov = Trust in Government; Own Risk = COVID-19 Concerns–Own Risk; Family = COVID-19 Concerns–Family Risk; Severity = COVID-19 Concerns–Severity; Adhere = Adherence; Diff adh = Adherence Difficulty; CVD Cons = COVID-19 Conspiracy Theories; Well = Threats–Wellbeing; Soc = Threats–Social; Mat = Threats–Material; Power = Power Distance; UA = Uncertainty Avoidance; Collect = Collectivism; LTO = Long-Term Orientation; Mas = Masculinity; Indulge = Indulgence; PT = Perspective-Taking; EC = Empathic Concern; PD = Personal Distress; NFC = Need for Cognition; React = Reactance; Conspir = Conspiracy Theory Beliefs; SD = Social Desirability.

#### COVID-19 diagnoses

Of the 332 respondents, 69 (20.78%) had been tested for COVID-19. A third of the sample (33.43%, *n* = 111) had an acquaintance who had been diagnosed with COVID-19 (*M*_severity rating_ = 3.32, *SD* = 1.44), with 23.19% (*n* = 77) indicating a family member, friend, or loved one has been diagnosed with the virus (*M*_severity rating_ = 3.35, *SD* = 1.40).

#### Self-reported adherence to restrictions

Respondents indicated that they adhered to COVID-19 restrictions recommended in their state (*M* = 4.55, *SD* = .69). For mask wearing, most respondents indicated that they wore a mask in public (*n*s = 230 always, 81 very often, 15 sometimes, 4 rarely, 2 never). Respondents indicated that they engaged moderately with media related to COVID-19 (*M* = 3.86, *SD* = 0.98). Respondents did not indicate resistance to government directives (“I don’t like the government telling me what to do about COVID-19 [e.g., that I must wear a mask in public, where I can go]”, *M* = 1.94, *SD* = 1.06).

#### Political views

Political party affiliation was weighted more towards Democrat (*n* = 154, 46.39%), followed by Republican (*n* = 86, 25.90%), Independent (*n* = 77, 23.19%), and Undecided/Other (*n* = 15, 4.52%). Respondents characterized themselves between progressive and conservative when it came to their political views about social (*M* = 3.44, *SD* = 1.82) and economic (*M* = 3.71, *SD* = 1.79) issues.

Overall, respondents were dissatisfied with then President Trump’s handling of COVID-19 in the United States (*M* = 2.19, *SD* = 1.36) with 47.89% (*n* = 159) of respondents very dissatisfied, 15.36% (*n* = 51) dissatisfied, 12.95 (*n* = 43) neither dissatisfied nor satisfied, 17.47% (*n* = 58) satisfied, and 6.33% (*n* = 21) very satisfied. Examination of satisfaction by political party affiliation using the Kruskal-Wallis *H* Test revealed that approval of then President Trump’s COVID-19 response was significantly different by political party, *H*(3) = 108.42, *p* < .001. Pairwise comparisons with Bonferroni adjusted *p*-values showed that were significant differences (at *p* < .001) on approval between Republicans (*Mdn* = 4) and Democrats (*Mdn* = 1); between Republicans and Independents (*Mdn* = 2); between Republicans and Undecided/Other (*Mdn* = 1); and between Democrats and Independents.

### Predicting attitudes to restrictions

General attitudes to easing COVID-19 restrictions and attitudes to easing specific COVID-19 restrictions were the dependent variables in a series of multiple regression analyses. Given the large number of variables and mainly exploratory nature of the study, a series of regressions were conducted with separate regressions conducted for blocks of variables: demographics, political opinions, experiences of COVID-19, cultural dimension variables, and other individual differences variables. Social desirability was included in all analyses (except demographics) in Step 1 of the analysis given correlations between this variable and attitudes to easing general restrictions, as well as other demographic variables. Tables 10–14 present for each regression analysis the variance explained by the model (*R*^*2*^), the change in variance (*ΔR*^*2*^) explained for models where social desirability was included at Step 1 and all other predictors at Step 2, and the significance (*p*) of these values; and for individual predictors, the unstandardized betas (*b*), 95% confidence intervals (95% CI), standard errors of *b* (*SE*_*B*_), standardized beta coefficients (*β*), and significance values (*p*) are included. Demographic variables explained a small amount of population variance (*R*^*2*^) for both general (6%) and specific (7%) attitudes to easing restrictions. For both measures, being a college graduate was associated with greater general (*β* = .16) and specific (*β* = .17) support for easing restrictions. For general attitudes to restrictions, respondents of Asian ethnicity were less likely to support general lifting of restrictions compared with White (the reference variable in the regression) respondents (*β* = -.12); for specific easing of restrictions, being female (*β* = -.11) and older (*β* = -.13) were associated with lesser support for easing restrictions.

**Table 10:** Multiple regression for predicting attitudes to easing COVID-19 restrictions for demographic factors.

	*General attitudes*					*Specific attitudes*				
	*b*	95% CI	*SE*_*B*_	*β*	*Sig*.	*b*	95% CI	*SE*_*B*_	*β*	*Sig*.
Constant	23.12	(20.76, 25.47)	1.20		< .001	36.51	(32.97, 40.05)	1.80		< .001
Gender	-2.03	(-4.14, 0.09)	1.07	-.10	.06	-3.26	(-6.44, -0.09)	1.62	-.11	.04
Age	-0.003	(-0.01, 0.003)	0.003	-.05	.36	-0.01	(-0.02, -0.001)	0.004	-.13	.02
Black ethnicity	-0.96	(-4.00, 2.08)	1.54	-.04	.54	-4.51	(-9.08, 0.06)	2.32	-.11	.05
Asian ethnicity	-4.35	(-8.24, -0.46)	1.98	-.12	.03	-4.07	(-9.92, 1.79)	2.97	-.08	.17
Other (incl. Hispanic)	2.50	(-1.21, 6.21)	1.89	.07	.19	2.39	(-3.20, 7.97)	2.84	.05	.40
Household income	0.55	(-1.71. 2.80)	1.14	.03	.63	-0.02	(-3.40, 3.37)	1.72	-.001	.99
College graduate	3.38	(1.03, 5.72)	1.19	.16	.01	5.48	(1.94, 9.01)	1.80	.17	.002

*Note*: *R*^*2*^ *General* = .06 (*p* = .01). *R*^*2*^ *Specific* = .07 (*p* = .001). Gender: 0 = Male, 1 = Female. Ethnicity: 0 = No, 1 = Yes. Household income: 0 = less than $59,000, 1 = $60,000+. College graduate: 0 = No, 1 = Yes.

**Table 11:** Multiple regression for predicting attitudes to easing COVID-19 restrictions for political views.

	*General attitudes*					*Specific restrictions*				
	*b*	95% CI	*SE*_*B*_	*β*	*Sig*.	*b*	95% CI	*SE*_*B*_	*β*	*Sig*.
Step 1										
Constant	18.45	(13.01, 23.89)	2.77		< .001	30.00	(21.76, 38.23)	4.19		< .001
Social desirability	0.14	(0.01, 0.26)	0.07	.11	.04	0.17	(-0.03, 0.36)	0.10	.09	.09
Step 2										
Constant	11.21	(4.59, 17.84)	3.37		< .001	17.07	(6.83, 27.31)	5.21		< .001
Social desirability	0.07	(-0.04, 0.18)	0.06	.06	.22	0.08	(-0.09, 0.26)	0.09	.05	.34
Democrat	-3.58	(-6.60, -0.56)	1.54	-.18	.02	-3.28	(-7.95, 1.39)	2.37	-.11	.17
Independent	0.54	(-2.41, 3.48)	1.50	.02	.72	3.00	(-1.55, 7.56)	2.31	.09	.20
Undecided/other	-1.71	(-6.65, 3.22)	2.51	-.04	.50	1.24	(-6.39, 8.87)	3.88	.02	.75
Social conservatism	0.98	(0.05, 1.91)	0.47	.18	.04	1.77	(0.33, 3.21)	0.73	.22	.02
Economic conservatism	1.43	(0.46, 2.39)	0.49	.26	.004	1.98	(0.49, 3.48)	0.76	.24	.01
Trust in Government	0.10	(-0.01, 0.20)	0.06	.08	.08	0.12	(-0.04, 0.29)	0.08	.07	.15

*Note*: *General–R*^*2*^ for Step 1 = .01 (*p* = .04), Δ*R*^*2*^ for Step 2 = .29 (*p* < .001). *Specific–R*^*2*^ for Step 1 = .01 (*p* = .09), Δ*R*^*2*^ for Step 2 = .26 (*p* < .001). Political affiliation: 0 = No, 1 = Yes.

**Table 12:** Multiple regression for predicting attitudes to easing COVID-19 restrictions for experiences.

	*General attitudes*					*Specific attitudes*				
	*b*	95% CI	*SE*_*B*_	*β*	*Sig*.	*b*	95% CI	*SE*_*B*_	*β*	*Sig*.
Step 1										
Constant	19.93	(14.50, 25.37)	2.76		< .001	32.44	(24.25, 40.64)	4.16		< .001
Social desirability	0.09	(-0.04, 0.22)	0.07	.08	.17	0.10	(-0.10, 0.29)	0.10	.05	.33
Step 2										
Constant	31.33	(19.72, 42.95)	5.90		< .001	47.61	(29.62, 65.61)	9.14		< .001
Social desirability	0.04	(-0.07, 0.14)	0.05	.03	.48	0.02	(-0.15, 0.18)	0.08	.01	.84
Family/friend diagnosed	-1.79	(3.80, 0.23)	1.03	-.08	.08	-3.11	(-6.24, 0.01)	1.59	-.09	.05
Acquaintance diagnosed	-0.89	(-2.65, 0.88)	0.90	-.04	.32	1.29	(-1.45, 4.03)	1.39	.04	.35
Concerns About Family	-0.32	(0.62, -0.02)	0.15	-.12	.04	-0.38	(-0.84, 0.09)	0.24	-.09	.11
Concerns of Own Risk	0.03	(-0.26. 0.32)	0.15	.01	.85	-0.06	(-0.51, 0.39)	0.23	-.02	.80
Concerns of own COVID-19 severity	-0.44	(-0.86, -0.02)	0.21	-.12	.04	-0.47	(-1.11, 0.18)	0.33	-.09	.16
Wellbeing threat	-0.19	(-0.30, -0.08)	0.06	-.25	< .001	-0.30	(-0.47, -0.13)	0.09	-.26	< .001
Social threat	0.17	(0.10, 0.25)	0.04	.27	< .001	0.25	(0.14, 0.37)	0.06	.26	< .001
Material threat	-0.001	(-0.07, 0.07)	0.04	-.002	.97	-0.04	(-0.15, 0.07)	0.06	-.04	.52
Adherence to restrictions	-0.83	(-1.44, -0.22)	0.31	-.14	.01	-1.17	(-2.12, -0.23)	0.48	-.13	.02
Difficulty in adherence to restrictions	0.12	(-0.27, 0.52)	0.20	.03	.54	0.22	(-0.39, 0.83)	0.31	.04	.48
COVID-19 Conspiracy Theories	0.49	(0.33, 0.66)	0.08	.28	< .001	0.65	(0.39, 0.90)	0.13	.25	< .001
Not liking government directives	1.91	(0.91, 2.91)	0.51	.20***	< .001	3.54	(1.99, 5.09)	0.79	.24	< .001

*Note*: *General–R*^*2*^ for Step 1 = .01 (*p* = .17), Δ*R*^*2*^ for Step 2 = .48 (*p* < .001). *Specific–R*^*2*^ for Step 1 = .003 (*p* = .33), Δ*R*^*2*^ for Step 2 = .45 (*p* < .001).

**Table 13:** Multiple regression for predicting attitudes to easing COVID-19 restrictions for cultural dimensions.

	*General attitudes*					*Specific attitudes*				
	*b*	95% CI	*SE*_*B*_	*β*	*Sig*.	*b*	95% CI	*SE*_*B*_	*β*	*Sig*.
Step 1										
Constant	18.06	(12.58, 23.53)	2.78		< .001	29.29	(21.03, 37.56)	4.20		< .001
Social desirability	0.14	(0.01, 0.27)	0.07	.12*	.03	0.18	(-0.01, 0.38)	0.10	.10	.07
Step 2										
Constant	16.78	(6.49, 27.07)	5.23		.001	30.34	(14.37, 46.31)	8.12		< .001
Social desirability	0.12	(0.001, 0.23)	0.06	.10	.05	0.15	(-0.03, 0.33)	0.09	.08	.11
Power distance	0.31	(-0.01, 0.63)	0.16	.12	.05	0.33	(-0.16, 0.83)	0.25	.08	.19
Uncertainty avoidance	-0.56	(-0.98, -0.14)	0.22	-.15**	.01	-0.97	(-1.62, -0.32)	0.33	-.17	.004
Collectivism	-0.45	(-0.67, -0.22)	0.11	-.22***	< .001	-0.53	(-0.88, -0.18)	0.18	-.17	.003
Long-term Orientation	0.36	(0.03, .68)	0.17	.12*	.03	0.28	(-0.23, 0.78)	0.26	.06	.29
Masculinity	0.94	(0.64, 1.23)	0.15	.38***	< .001	1.32	(0.86, 1.78)	0.23	.35	< .001
Indulgence	0.20	(-0.31, 0.71)	0.26	.04	.43	0.72	(-0.07, 1.52)	0.40	.10	.07

*Note*: *General–R*^*2*^ for Step 1 = .01 (*p* < .05), Δ*R*^*2*^ for Step 2 = .25 (*p* < .001). *Specific–R*^*2*^ for Step 1 = .01 *(*p >* .05), Δ*R*^*2*^ for Step 2 = .21 (*p* < .001).

**Table 14:** Multiple regression for predicting attitudes to easing COVID-19 restrictions for individual differences factors.

	*General attitudes*					*Specific attitudes*				
	*b*	95% CI	*SE*_*B*_	*β*	*Sig*.	*b*	95% CI	*SE*_*B*_	*β*	*Sig*.
Step 1										
Constant	18.47	(13.01, 23.92)	2.77		< .001	30.11	(21.86, 38.36)	4.19		< .001
Social desirability	0.14	(0.01, 0.26)	0.07	.11	.04	0.17	(-0.03, 0.36)	0.10	.09	.10
Step 2										
Constant	17.10	(6.29, 27.91)	5.49		.002	27.86	(11.76, 43.97)	8.19		< .001
Social desirability	0.22	(0.07, 0.37)	0.08	.18	.004	0.34	(0.12, 0.56)	0.11	.19	.003
Perspective-Taking	-0.21	(-0.48, 0.07)	0.14	-.09	.15	-0.19	(-0.60, 0.22)	0.21	-.06	.36
Empathic Concern	-0.20	(-0.44, 0.05)	0.13	-.10	.12	-0.39	(-0.76, -0.02)	0.19	-.13	.04
Personal Distress	-0.24	(-0.44, -0.03)	0.11	-.13	.02	-0.46	(-0.77, -0.15)	0.16	-.17	.003
Need for Cognition	-0.07	(-0.27, 0.13)	0.10	-.04	.49	-0.27	(-0.58, 0.03)	0.16	-.10	.08
Reactance	0.08	(-0.06, 0.23)	0.07	.07	.27	0.26	(0.05, 0.48)	0.11	.14	.02
Conspiracy Theory Beliefs	0.20	(0.13, 0.28)	0.04	.29	< .001	0.28	(0.16, 0.39)	0.06	.26	< .001

*Note*: *General–R*^*2*^ for Step 1 = .01 (*p* = .04), Δ*R*^*2*^ for Step 2 = .14 (*p* < .001). *Specific–R*^*2*^ for Step 1 = .01 (*p* = .09), Δ*R*^*2*^ for Step 2 = .17 (*p* < .001).

Political views explained 31% (general) and 27% (specific) of population variance. In both general and specific models, greater social (*β* = .18 & *β* = .22) and economic conservatism (*β* = .26 & *β* = .24) were associated with greater support for easing restrictions. Being a Democrat, compared to a Republican (the reference variable in the regression), was associated with lesser general support for easing restrictions (*β* = -.18).

Experiences of COVID-19 explained 49% (general) and 46% (specific) of population variance in attitudes to easing restrictions. For both general and specific attitudes, greater belief in conspiracy theories related to COVID-19 (*β* = .28 & *β* = .25) and greater resistance to government directives (*β* = .20 & *β* = .24) were associated with greater support for easing restrictions. Greater wellbeing threat was associated with lesser support for easing restrictions at both levels (*β* = -.25 & *β* = -.26). By contrast, greater concern about social impact of COVID-19 was associated with greater support for easing restrictions at both levels (*β* = .27 & *β* = .26). Greater concern for family members (*β* = -.12) and greater concerns about own severity if infected (*β* = -.12) were associated with lesser general support in for easing restrictions. Finally, greater adherence to restrictions was associated with lesser support for easing restrictions (*β* = -.14 & *β* = .13).

Cultural values predicted 26% (general) and 22% (specific) of population variance in attitudes to easing restrictions. In both models, greater uncertainty avoidance (*β* = -.15 & *β* = -.17) and collectivism (*β* = -.22 & *β* = -.17) were associated with lesser support for easing restrictions; greater masculinity was associated with greater support (*β* = -.38 & *β* = -.35). For general attitudes, greater long-term orientation was associated with greater support for easing restrictions (*β* = .12).

Individual difference factors predicted 15% (general) and 18% (specific attitudes) of population variance, with greater conspiracy theory beliefs in both models associated with greater support for easing restrictions (*β* = .29 & *β* = .26). Only personal distress was associated with general attitudes to easing restrictions (*β* = -.13), with greater personal distress associated with lesser support for easing restrictions. For specific easing of restrictions, greater empathic concern was associated with lesser support (*β* = -.13), and greater psychological reactance (*β* = .14) with greater support. Greater social desirability concerns predicted greater support for easing restrictions in both models.

## Discussion

**Fig 1:**
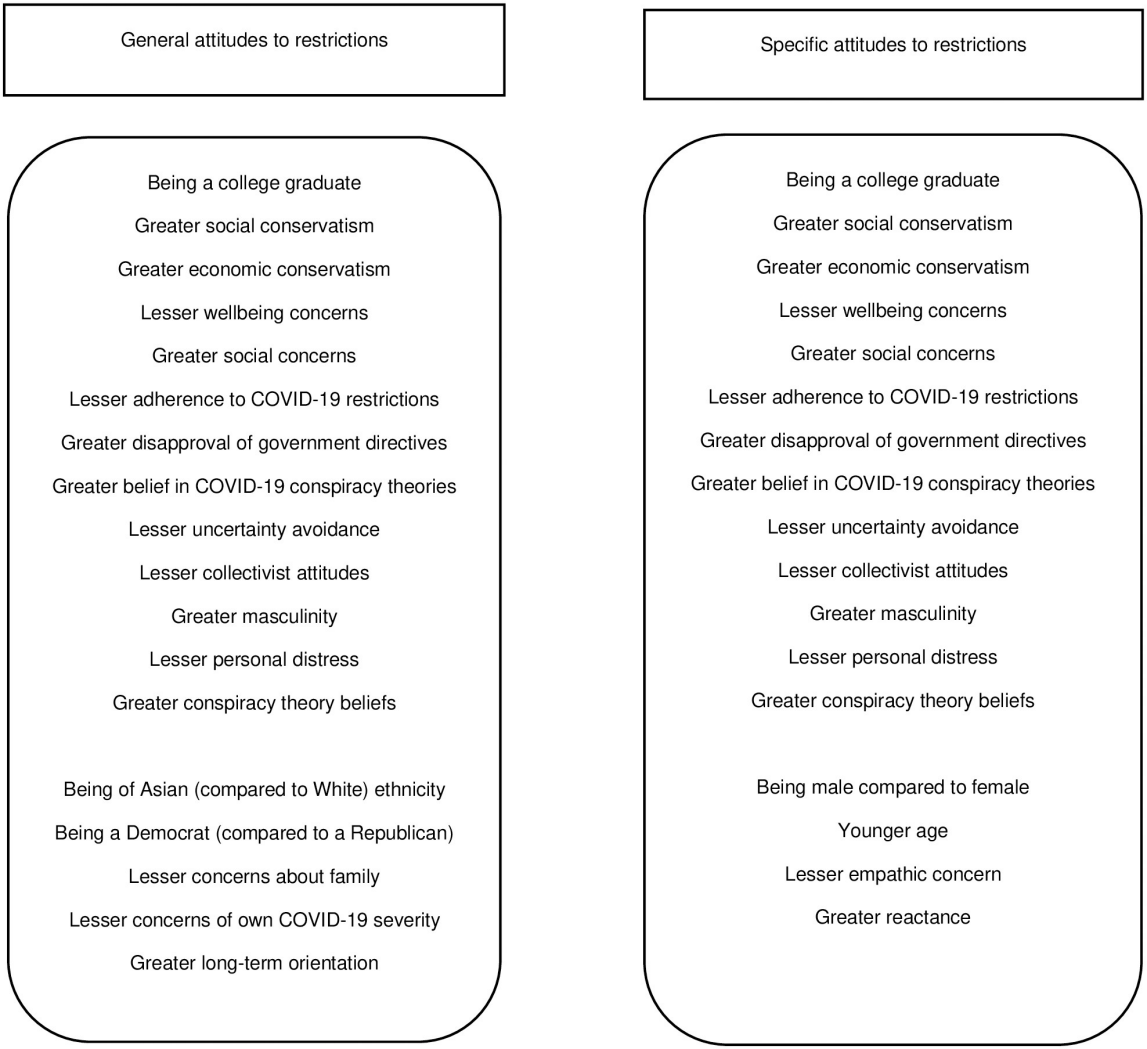
Significant predictors of greater support for easing COVID-19 restrictions for general and specific attitudes.

The purpose of this study was to investigate factors that influence United States’ citizens support or opposition for easing COVID-19 restrictions. Several factors emerged as predictors of both *general* attitudes to easing restrictions and attitudes to easing *specific* measures, and most were in line with our hypotheses. Significant predictors that involved respondents’ experiences and perceptions of COVID-19 involved perceived risk of the virus to the health of oneself and family, perceived threats posed by the virus, beliefs in conspiracy theories related to the virus, and reluctance to follow directives from the government regarding transmission reduction strategies (e.g., wearing masks, limits on travel). Political views were also important, with political affiliation (Democrat vs. Republican) and social and economic conservatism predicting attitudes to easing restrictions. Cultural orientation and individual difference factors, mainly those involving degree of consideration of others (collectivism, masculinity, empathic concern, personal distress), as well as long-term orientation, avoidance of uncertainty, reactance, and general conspiracy theory beliefs significantly predicted either general or specific attitudes. For demographic factors, education, ethnicity (Asian [vs. White] ethnicity), gender, and age helped explain attitudes to restrictions. Fig 1 presents the significant predictors for both general and specific attitudes to restrictions.

Findings point to the importance of both proximal (e.g., experience) and distal (e.g., individual differences) factors in understanding reluctance towards or support of easing restrictions. Specifically, increased motivation and confidence to adhere to restrictions, concern about the effects on family members of COVID-19, perceptions of how severe the virus would be for oneself, and perceptions of threats to one’s health and wellbeing, predicted lesser support for easing of COVID-19 restrictions. Such findings are consistent with explanations of how individuals enact health behaviors [20–22, 25], of which adhering to (or wanting continued) restrictions could be seen as a protective behavior against threat of the virus, like other studied COVID-19 limiting behaviors [28]. Furthermore, findings are consistent with health behavior models that stress the importance of self-efficacy and perceptions that one’s behaviors will be effective in addressing threat [29].

Respondents who felt that their physical and mental health was compromised by COVID-19 were more reluctant for restrictions to be eased. The scale used to measure this (the health and wellbeing subscale of the *COVID-19 Multifaceted Threat Scale*; [77]) measures both physical health concerns (e.g., catching the virus) and concerns regarding mental health, such as worries about isolation. There has been discussion of the effects on mental health of sustained lockdowns as justification to ease restrictions [88, 89]. For respondents, it may be that health concerns overrode a want to end deleterious effects such as isolation, or that respondents saw restrictions as a way to get back to ‘normal’. Indeed, at the time of data collection (in mid-2020), the idea of complete elimination of the virus may have been seen by respondents as a possibility, with restricting transmission now seen as a more sustainable goal (see [90]).

Greater belief in COVID-19 conspiracy theories predicted greater support for easing restrictions. In the present study, a measure was devised that largely focused on conspiracy theories relating to the origin of the disease. This measure was very highly correlated with more general beliefs in conspiracy theories, which was also a significant predictor of wanting to ease restrictions. This suggests that individuals inclined to believe more generally that world events are controlled by powerful people and groups also interpret the causes of COVID-19 from a similar vantage point. Findings extend work conducted examining attitudes towards COVID-19 restrictions and trust of authorities, such as politicians and health professionals [39]. Indeed, both being averse to being ‘told what to do’ by authorities (specifically related to COVID-19) and trait psychological reactance significantly predicted support for easing restrictions in the present study.

Demographic factors were important in understanding attitudes to easing restrictions. College-educated respondents were more supportive of easing of restrictions. Results regarding education are mixed in the previous literature. For example, higher levels of education have predicted increased engagement in preventative behaviors (e.g., avoiding public places or public transport; [76]), but not frequency of engagement (e.g., of washing hands, wearing a mask, social distancing [91]). In addition, education did not significantly predict beliefs in necessity of COVID-19-related public health initiatives [74]. Gender (being female) and older age predicted lesser support for easing restrictions in the present study. Studies across countries have found that females are more likely to agree to comply with restrictions [92]. Age is a somewhat complex correlate of COVID-19 behaviors, with concerns raised about compliance with directives by older persons [93]. In the present study, age was associated with greater support for maintaining restrictions, perhaps due to perceived vulnerability related to health concerns [74], or a more compliant group of respondents, as evidenced by adherence to COVID-19 protective behaviors such as mask wearing. Interestingly, each of these findings pertaining to demographic factors are consistent with previous studies exploring demographic and attitudinal determinants of protective behaviors during the SARS pandemic [20]. That Asian ethnicity emerged as a predictor of greater support for restrictions likely reflects concerns or fears due to anti-Asian sentiment following the virus’ initial identification in China [94, 95]. Notably, anti-Asian fear during the pandemic has been associated with more conservative political affiliation [95].

Political affiliation and degree of liberal versus conservative orientation regarding social and economic issues predicted support or opposition to easing restrictions. Those who identified as Democrats (compared with Republicans) were most supportive of maintaining restrictions, while more economically and socially conservative respondents supported easing of restrictions. The link between political affiliation or ideology and attitudes to COVID-19-related information has been debated. Some authors relate it to statements made by (then) President Trump and others downplaying the virus and consensus seeking by wanting to hold similar attitudes to one’s political party [96]. Other perspectives point to political ideologies associated with conservatism, such as not wanting increased government restriction (e.g., through lockdowns), which then influences conservatives to downplay the threat posed by the disease [38]. Indeed, the finding here that greater economic conservativism was associated with wanting to ease restrictions suggests values around maintaining the economy and protecting business interests.

Several individual difference factors predicted attitudes to easing restrictions. Both greater masculinity and long-term orientation predicted greater support for easing lockdowns. Masculinity, particularly defined by Hofstede, involves a focus on equity, competition, strength, and economic growth, with more feminine ideals considered to focus on equality, solidarity, and welfare assistance [97]. Many of these attributes may explain a response focusing on reopening, with lesser concern for the welfare of needy others. Indeed, masculinity has been associated with lesser empathy and prosocial behavior [98, 99]. Authors specifically focusing on COVID-19 have suggested that aggressive masculinity has a role to play in ideas of post-truth and misinformation [100]. Somewhat surprisingly, long-term orientation predicted wanting to ease restrictions. The focus of this orientation on longer term goals [43] would plausibly suggest that individuals may realize that longer periods of time under restrictions will be required to return to a world that is closer to pre-pandemic. Long-term orientation has been little investigated regarding health decisions, but the concept does stress thrift and has been applied to more tempered decision making regarding financial decisions [53]. Considering COVID-19 specifically, respondents in the present study who were higher on this orientation may have felt that there is a need to move towards easing restrictions in a situation that is likely to be of concern for an extended period both in the United States and worldwide.

Greater uncertainty avoidance, collectivism, and empathy (empathic concern and personal distress) predicted lesser support for easing restrictions when considered with other individual difference or cultural factors. Avoidance of uncertainty likely reflects the considerable uncertainty posed by COVID-19 and a resulting want for preventative measures and caution. Previous work has found that when individuals who are higher on uncertainty avoidance perceive a health message as credible, they are more likely to comply [101]). Respondents in the present study tended to be supportive of engaging in preventative measures and were generally supportive of restrictions. The emergence of greater collectivism, empathic concern, and personal distress as predictors of greater reluctance to ease restrictions suggests that those sensitive to and apt to consider others in decision making, such as the effects of COVID-19 on family or community, support preventative health approaches. Empathic concern is a predictor of helping behavior [57], and so respondents may perceive adhering to restrictions as helping others, such as family members and loved ones. The significant predictive power of personal distress, which is an empathic response to others but involves anxiety that is more self-oriented, perhaps indicates the presence of anxiety leading to wanting to avoid risk and implement control mechanisms such as maintaining restrictions.

It is important to acknowledge that many of the investigated factors are interrelated. While there was not an underlying theoretical hypothesis that focused on a small number of predictors, the consideration of both proximal (e.g., perceived risk) and distal (e.g., individual difference) factors is likely to provide a more nuanced picture of attitudes and behaviors around COVID-19. That health, political, demographic, and personality dimensions had a role to play in understanding attitudes to restrictions suggests the complexity of decision-making around the pandemic. The investigation of perceptions of risk, experiences, and individual difference variables in relation to restrictions is also relevant for understanding attitudes to other COVID-19-reduction strategies. For example, while COVID-19 vaccines were not available at the time of data collection (and, therefore, not investigated), literature on understanding vaccination uptake has pointed to factors such as risk perception, trust, political orientation, and other factors investigated here including conspiracy theories [102, 103].

### Limitations

There were several limitations to this study that warrant consideration. First, respondents were recruited for diversity in key demographic factors. Despite this, the sample did tend to be politically liberal and more supportive of maintaining restrictions. Consideration of how observed relationships are similar or different with diverse samples is warranted. Second, the study was focused on the United States, where the gravity of the pandemic and considerable political and social unrest is heightened compared to other parts of the world. As such, circumstances of individual countries may drive decision-making in different ways, limiting the generalizability of findings. Third, owing to the novelty of the virus, new measures were created for use in this study that require further validation. Two measures of attitudes to lockdowns were presented (general vs. specific), which were quite highly correlated. However, there were some differences in strength of associations with independent variables and so both were included and may be used depending on future research purposes. Conceptualizing cultural dimensions from an individual perspective is also fairly novel (as opposed to the typical convention of measuring these dimensions at the national or cultural level), although many of the factors are conceptually similar to variables studied within psychology more broadly. Finally, given the unfolding situation, longitudinal consideration of attitudes to restrictions should be considered, as individuals become fatigued by sometimes frequent or long restrictions.

## Conclusion

In this study, perceptions of risk and concerns regarding COVID-19, political and ideological beliefs, and individual difference dimensions that underlie consideration of others, predicted attitudes towards easing COVID-19 restrictions. Based on findings and emerging work in the area, couching restrictions in terms of their importance to protect oneself and others, building self-efficacy in adhering to protective measures, and focusing on increasing concern for others, while minimizing distress or defensive reactions, are vital. This will likely help policy makers and governments move forward both through continued restrictions, but also as countries emerge from restrictions or re-enter restrictions, requiring adaptation and resilience of citizens.
